# The IRE1 α -XBP1s-NF κ B axis controls cell survival and epithelial differentiation under osmotic stress through arachidonic acid metabolism activation

**DOI:** 10.15698/cst2026.04.317

**Published:** 2026-04-24

**Authors:** Leandro Gastón Parra, Cecilia Irene Casali, Dylan Ezequiel Sendyk, Ailén Florencia Salafia, Sabrina Andrea Flor, Silvia Edith Lucangioli, María del Carmen Fernández Tome

**Affiliations:** 1 Universidad de Buenos Aires, Facultad de Farmacia y Bioquímica, Departamento de Ciencias Biológicas, Cátedra de Biología Celular y Molecular, Buenos Aires, Argentina; 2 Universidad de Buenos Aires, Facultad de Farmacia y Bioquímica, Departamento de Tecnología Farmacéutica, Buenos Aires, Argentina; 3 Universidad de Buenos Aires, Facultad de Farmacia y Bioquímica, Departamento de Ciencias Químicas, Buenos Aires, Argentina; 4 Universidad de Buenos Aires, Facultad de Farmacia y Bioquímica, Instituto de Tecnología Farmacéutica y Biofarmacia (InTecFyB), Buenos Aires, Argentina; 5 Consejo Nacional de Investigaciones Científicas y Técnicas, Instituto de Química y Fisicoquímica Biológicas Prof. Dr. Alejandro C. Paladini (IQUIFIB)-Facultad de Farmacia y Bioquímica, Buenos Aires, Argentina; 6 Consejo Nacional de Investigaciones Científicas y Técnicas (CONICET), Godoy Cruz 2290, Buenos Aires, Argentina

**Keywords:** arachidonic acid, phospholipase A2, cyclooxygenase 2, ER stress, unfolded protein response, osmotic stress, cell differentiation

## Abstract

Arachidonic acid (AA) metabolism plays a critical role in renal cell osmoadaptation. We recently demonstrated that hypertonicity induces the expression and activation of cytosolic phospholipase A ${}_{2}$ (cPLA ${}_{2}$ ). On one hand, AA released by cPLA ${}_{2}$ enhances triacylglyceride (TG) synthesis and accumulation. On the other hand, AA is converted into prostaglandins (PG) through cyclooxygenase 2 (COX2) activity. Both processes are required for renal cell survival under osmotic stress. However, the mechanisms by which hypertonicity induces cPLA ${}_{2}$ expression remain poorly understood. Given that we previously shown that hypertonicity regulates TG synthesis through the IRE1 α -XBP1s branch of the unfolded protein response (UPR), here we examined whether XBP1s regulates the cPLA ${}_{2}$ -AA-COX2 axis in renal cells subjected to osmotic stress. We found that XBP1s modulates hypertonicity-induced expression of cPLA ${}_{2}$ and COX2 by increasing NF κ B transcriptional activity. Inhibition of IRE1 α impaired normal COX2 degradation and disrupted AA metabolism, leading to a decrease in cell viability and preventing hypertonicity-induced epithelial differentiation. Prostaglandin E2 (PGE2) contributed to cell polarization facilitating adherens junction (AJ) assembly. Together, these findings highlight a central role for the IRE α -XBP1s-NF κ B signaling axis in coordinating cell stress responses and epithelial differentiation through AA metabolism activation.

## INTRODUCTION

Renal medullary cells are physiologically surrounded by an unique extracellular matrix (ECM) characterized by the presence of an osmolarity gradient along the cortico-medullary axis. In the renal cortex, osmolality is around 290 mOsm/kg of H ${}_{2}$ O and reaches 800–1200 mOsm/kg of H ${}_{2}$ O in the tip of the inner medulla. Moreover, ECM osmolality is even higher under water restriction 1, 2. This high medullary interstitium osmolarity is generated by the accumulation of NaCl and urea, and is necessary for both, the urine-concentrating mechanism, which is required to maintain hydro-saline corporal equilibrium, and the differentiation of the medullary tubular structures during kidney development. Since the medullary cells are constantly exposed to abrupt changes in ECM osmolarity, they must implement a complex adaptation program to survive in this environment.

Our laboratory extensively studied how lipid metabolism contributes to osmoprotection. We demonstrated that glycerolipid biosynthesis and membrane turnover are protective mechanisms against osmotic changes; when environmental osmolarity increases, the biosynthesis of fatty acids (FA) as well as glycerophospholipids (GP) and triacylglycerides (TG) are rapidly upregulated 3, 4. These biosynthetic pathways allow the renewal and expansion of the cellular membranes; this is especially important for maintaining the endoplasmic reticulum (ER) homeostasis, since the augmentation of membrane area contributes to alleviate the ER stress caused by the high synthesis of membrane-associated osmoprotective proteins and saturated FA as well 4, 5.

Hypertonicity-induced ER stress and the subsequent activation of the UPR play a protective role during renal osmoadaptation. UPR signaling decreases the expression of the epithelial sodium channel (ENaC) in the outer medulla of water-deprived mice, thereby contributing to the maintenance of body fluid homeostasis under conditions of dehydration 6. In addition, we showed that hypertonicity-induced ER stress in the renal epithelial cell line Madin-Darby canine kidney (MDCK) activates the IRE1 α -XBP1s UPR pathway which controls GP synthesis by regulating the expression and activity of lipogenic enzymes and transcription factors 7.

The hypertonic environment also regulates arachidonic acid (AA) metabolism. We and others demonstrated that renal cells subjected to hyperosmolarity upregulate the expression and activity of two key enzymes in the transformation of AA: cyclooxygenase 2 (COX2), a key enzyme in the conversion of AA into prostaglandins (PG), and cytosolic phospholipase A ${}_{2}$ (cPLA ${}_{2}$ ) that releases AA from membrane GP 8. Since the knockdown and/or inhibition of COX2 and/or PLA ${}_{2}$ decrease cell viability leading to cell death, both enzymes are considered cytoprotective proteins 9, 10. In renal medullary tissue we have shown that COX2-mediated PG synthesis regulates GP synthesis thus maintaining cellular membrane homeostasis 11. Likewise, we showed that the increase in environmental osmolarity activates TG synthesis and its storage in lipid droplets (LD) which are essential structures for maintaining cell membrane homeostasis and cell survival 4, 7. Regarding these findings we demonstrated that cPLA ${}_{2}$ -mediated AA release regulates TG synthesis and its storage through the activation of PPAR γ nuclear factor 10, highlighting the relevance of AA metabolism in cell survival. In addition to their role in lipid metabolism and cell osmoadaptation, PG mediate the recovery of renal epithelial cells from hyperoxaluria-induced damage. After calcium oxalate-induced injury, COX2-derived PGE2 mediates renal epithelial restitution, a process that requires cell redifferentiation 12. Although we have shown that hyperosmolarity activates the XBP1s branch of the UPR and AA metabolism, both implicated in the regulation of GL metabolism in renal epithelial cells, it remains unclear whether ER stress–UPR activation and AA metabolism are coordinated responses to increased environmental osmolarity. In this sense, Chopra et al showed that IRE1 α -XBP1s signaling controls PGE2 synthesis in leukocytes by modulating COX2 expression 13; however, the role of the IRE1 α -XBP1s pathway in hypertonicity-induced AA metabolism activation and its contribution to renal epithelial differentiation remain unknown.

Therefore, the aim of this study was to determine whether the IRE1 α -XBP1s pathway links hypertonicity-induced AA metabolism to osmoadaptation and renal epithelial differentiation. The data presented in this work indicate that hypertonicity-induced activation of XBP1s regulates AA metabolism by increasing NF κ B transcriptional activity which mediates the expression of cPLA ${}_{2}$ and COX2 proteins. XBP1s plays a fundamental role in cellular adaptation to osmotic stress: on one hand, it promotes the release of AA required to preserve membrane homeostasis and, on the other hand, it is required for epithelial differentiation by regulating PGE2 production, which in turn drives the assembly of adherens junction (AJ) complexes during the early stages of this process.

Together, these findings identify XBP1s as a key integrator of lipid metabolism and epithelial morphogenesis under hypertonic conditions and provide a potential target to preserve epithelial integrity in pathological states associated with altered renal medullary function. Our results demonstrate, for the first time, that the IRE1 α -XBP1s branch of the UPR plays a central role in the regulation of hypertonicity-induced AA metabolism and is required for both osmoadaptation and renal epithelial cell differentiation.

## RESULTS

### Hypertonicity-induced IRE1 α -XBP1s signaling leads to NF κ B transcriptional activation

**Figure 1:**
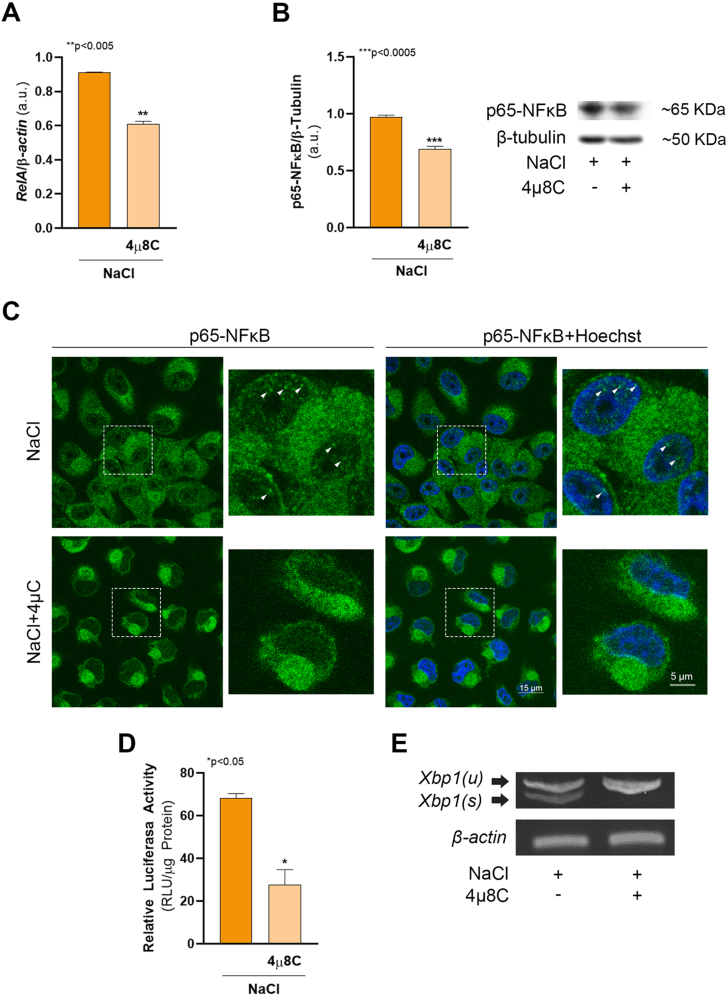
XBP1s regulates hypertonic-induced NF κ B transcriptional activity. MDCK cells were cultured as described in Cell culture section and incubated for 24 h in hypertonic medium (NaCl) in the presence or in the absence of 20 μ M 4 μ 8C. **(A)** After treatments, cells were subjected to RNA extraction and *RelA* mRNA expression levels were quantified by RT-qPCR. β *-actin* was used as housekeeping control. Results were expressed as the ratio between *RelA* and β *-actin* expression and represent the mean ± SEM of three independent experiments. **(B)** p65-NF κ B protein levels were determined by western blot. PVDF membranes were probed with mouse monoclonal anti p65-NF κ B (1:250) or mouse monoclonal anti β -Tubulin (1:1,000). The results were expressed as the ratio between p65-NF κ B and β -Tubulin (loading control) and represent the mean ± SEM of three independent experiments. Representative images of western blot obtained are shown. **(C)** MDCK cells were seeded on glass coverslips and cultured as described above. After treatments p65-NF κ B subcellular localization was determined by confocal microscopy using a monoclonal anti p65-NF κ B (1:50) primary antibody and a goat anti-mouse IgG Alexa Fluor 488-conjugated (1:200) secondary antibody (green). Nuclei were visualized with 2.5 μ M Hoechst 33258 (blue). Fluorescence microscopy images (630X magnification) were processed with Image J. White squares indicate the zones shown in the corresponding extended panels. White arrowheads indicate p65-NF κ B nuclear dots. Scale bars 15 μ m and 5 μ m in extended panels. **(D)** In another set of experiments, cells were grown up to 70% of confluence and transfected with 0.2 μ g of p κ B-Luc reporter plasmid. After 24 h cells incubated for 24 h in hypertonic medium (NaCl) in the presence or in the absence of 20 μ M 4 μ 8C. Luciferase activity was measured and the results were expressed as the ratio of luciferase activity units (RLU) and total protein content, and represents the mean ± SEM of three independent experiments. **(E)***Xbp1(s)* and *Xbp1(u)* RNA expressions were determined by RT-PCR, products were resolved in a 7% polyacrylamide gel containing ethidium bromide and visualized under UV light. Data were analyzed by unpaired two tailed Student’s t-test. ${}^{*}$ p < 0.05, ${}^{**}$ p < 0.005, ${}^{***}$ p < 0.005.

In a previous work we showed that hypertonicity induces ER stress and the IRE1 α -XBP1s pathway activation 7. Osmotic challenge also increases p65-NF κ B expression and activity 14. In addition, it was reported that ER stress induced-UPR activates NF κ B pathway through different molecular mechanisms 15. Thus, we studied whether the IRE1 α -XBP1s axis mediates NF κ B activation in cells subjected to hypertonic stress. To do this, we evaluated the effect of the inhibition of the IRE1 α RNAse activity on the expression, cellular distribution, and activity of p65-NF κ B protein (Figure 1). In cells pre-treated with 4 μ 8C, an IRE1 α RNAse activity inhibitor, the levels of *RelA* (encoding p65) and p65-NF κ B protein were significantly decreased (Figure 1**A** and **B**). When cellular distribution was evaluated by confocal microscopy (Figure 1**C**), we found that under hypertonic control conditions p65-NF κ B protein is distributed between both cytoplasmic and nuclear compartments, displaying a dotted pattern (white arrows, extended panel). In contrast, in cells pre-treated with 4 μ 8C, p65-NF κ B specific fluorescence was restricted to the cytoplasm even to a lesser extent and localized to large structures near nuclei. Consistent with these results, the transcriptional activity of p65-NF κ B measured by luciferase-gene reporter assay dropped by ∼ 60% (Figure 1**D**). Figure 1**E** confirms the action of 4 μ 8C on IRE1 α RNAse activity. These results demonstrate that *Xbp1* mRNA maturation is required for p65-NF κ B expression and activity, suggesting the possible role of XBP1s protein in the transcriptional regulation of *RelA*.

### IRE1 α -XBP1s-NF κ B signaling modulates cPLA ${}_{2}$ osmotic upregulation

**Figure 2:**
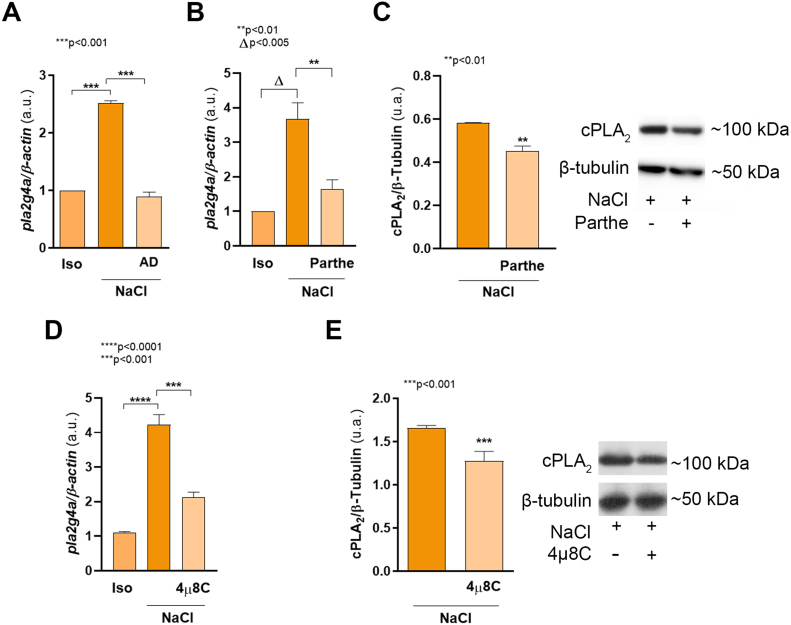
IRE1 α -XBP1s-NF κ B signaling modulates hypertonic-induced cPLA ${}_{2}$ upregulation. MDCK cells were cultured as described in Cell culture section and incubated for 24 h in isosmolar (Iso) or hypertonic (NaCl) medium in the presence or in the absence of 0.1 μ g/mL actinomycin D (AD) **(A)**, 3 μ M parthenolide (**B** and **C**) or 20 μ M 4 μ 8C (**D** and **E**). After treatments, cells were subjected to RNA extraction and *Pla2g4a* mRNA expression levels were quantified by RT-qPCR (**A, B** and **D**). β *-actin* was used as housekeeping control. Results were expressed as the ratio between *Pla2g4a* and β *-actin* expression and represent the mean ± SEM of three independent experiments. In other sets of experiments, cPLA ${}_{2}$ protein levels were determined by western blot (**C** and **E**). PVDF membranes were probed with mouse monoclonal anti cPLA ${}_{2}$ (1:250) or mouse monoclonal anti β -Tubulin (1:1,000). The results were expressed as the ratio between cPLA ${}_{2}$ and β -Tubulin (loading control) and represent the mean ± SEM of three independent experiments. Representative images of western blot obtained are shown. Data were analyzed by one-way ANOVA followed by a posteriori Dunnett’s test (**A**, **B** and **D**) or by unpaired two tailed Student’s t-test (**C** and **E**). ${}^{**}$ p < 0.01, Δ p < 0.005, ${}^{***}$ p < 0.001, ${}^{****}$ p < 0.0001.

It has been reported that the main regulation of cPLA ${}_{2}$ activity occurs at post-transcriptional level 16. However, the regulatory region of the *Pla2g4a* gene, which encodes cPLA ${}_{2}$ protein, contains response elements (RE) for different transcription factors, including NF κ B 17. During the adaptation period to osmotic changes (24 h), renal epithelial cells increase *Pla2g4a* mRNA levels, suggesting an osmotically induced transcriptional regulation. This was confirmed by pre-treating cells with the transcription inhibitor actinomycin D (AD, Figure 2**A**). Thus, we evaluated whether NF κ B is involved in hypertonic-induced cPLA ${}_{2}$ upregulation. To that end, MDCK cells were pre-treated with parthenolide (Parthe), an inhibitor of I κ B kinase and I κ B α phosphorylation that disrupts NF κ B activation 18. Parthe significantly reduced both *Pla2g4a* mRNA (Figure 2**B**, p < 0.01) and cPLA ${}_{2}$ protein levels (Figure 2**C**, p < 0.005). Consistent with the results shown in Figure 1, 4 μ 8C pre-treatment also reduced *Pla2g4a* mRNA and cPLA ${}_{2}$ protein expression (Figure 2**D**, p < 0.0002 and **E**, p < 0.001). These results show that p65-NF κ B mediates osmotic-induced cPLA ${}_{2}$ , suggesting the involvement of IRE1 α -XBP1s-NF κ B signaling pathway.

### IRE1 α -XBP1s-NF κ B signaling regulates the expression of the cytoprotective gene COX2

**Figure 3:**
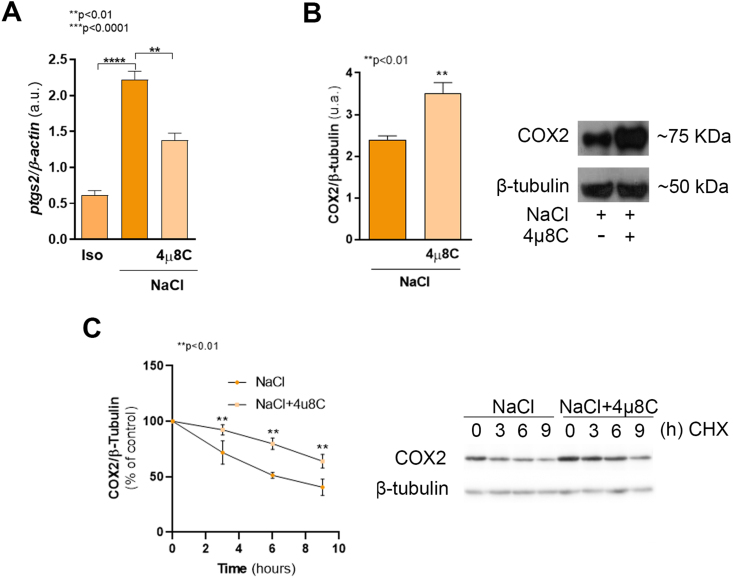
IRE1 α -XBP1s-NF κ B axis controls COX2 expression under osmotic stress. MDCK cells were cultured as described in Cell culture section and incubated for 24 h in isosmolar (Iso) or hypertonic (NaCl) medium in the presence or in the absence of 20 μ M 4 μ 8C. **(A)** After treatments, cells were subjected to RNA extraction and *Ptgs2* mRNA expression levels were quantified by RT-qPCR. β *-actin* was used as housekeeping control. Results were expressed as the ratio between *Ptgs2* and β *-actin* expression and represent the mean ± SEM of three independent experiments. **(B)** COX2 protein levels were determined by western blot. PVDF membranes were probed with rabbit polyclonal anti COX2 (1:250) or mouse monoclonal anti β -Tubulin (1:1,000). The results were expressed as the ratio between COX2 and β -Tubulin (loading control) and represent the mean ± SEM of three independent experiments. **(C)** After treatments, 25 μ M cycloheximide (CHX) was added to the culture medium and COX2 expression was determined by western blot at 0, 3, 6 and 9 h after protein synthesis inhibition. Results were expressed as % of remaining COX2 expression (COX2/ β -Tubulin ratio) relative to time 0 and represent the mean ± SEM of three independent experiments. Representative images of western blot obtained are shown. Data were analyzed by one-way ANOVA followed by a posteriori Dunnett’s **(A)** or Sidak’s test **(C)** or by unpaired two tailed Student’s t-test **(B)**. ${}^{**}$ p < 0.01, ${}^{***}$ p < 0.0001.

**Figure 4:**
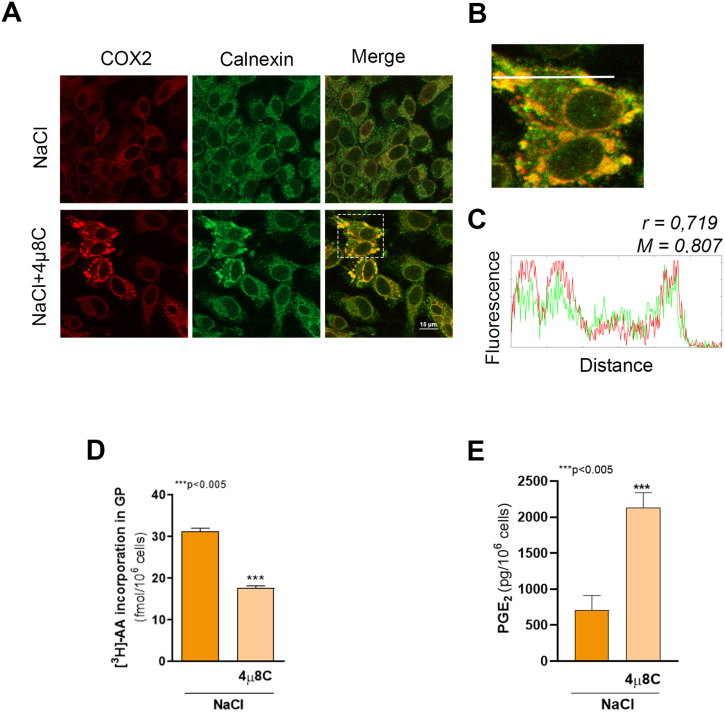
XBP1s downregulation results in COX2 accumulation within the ER. MDCK cells were cultured as described in Cel culture section and incubated for 24 h in hypertonic medium (NaCl) in the presence or in the absence of 20 μ M 4 μ 8C. **(A)** After treatments COX2 subcellular localization was determined by confocal microscopy using a rabbit polyclonal anti-COX2 (1:75) and donkey anti-rabbit (1:200) Alexa Fluor 546-conjugated antibody (red). Mouse monoclonal anti-Calnexin (1:50) and goat anti-mouse IgG Alexa Fluor 488-conjugated (1:200) secondary antibody were used to visualize ER (green). Fluorescence microscopy images (630X magnification) were processed with Image J. Scale bar 15 μ m. White square represents the region of interest (ROI) in which colocalization analysis was performed. **(B)** Extended panel of ROI, white line represents the region where line profile analysis was performed. **(C)** Line profile analysis. Pearson correlation coefficient (r) and overlapping Manders coefficient (M) calculated in the ROI are indicated. **(D)** In other experiments, three hours before harvesting, 0.1 μ Ci/mL of ${}^{3}$ H-AA were added to each well and after labeling incorporation of ${}^{3}$ H-AA to GP was determined. Results are expressed as fmol of ${}^{3}$ H-AA-GP per 10 ${}^{6}$ cells and represents the mean ± SEM of three independent experiments. **(E)** After treatments, culture media were collected, PG were extracted and the content of PGE ${}_{2}$ was determined by HPLC-MS/MS. Results were expressed as pg of PGE2 per 10 ${}^{6}$ cells and represents the mean ± SEM of three independent experiments. Data were analyzed by unpaired two tailed Student’s t-test. ${}^{***}$ p < 0.005.

Hypertonicity upregulates COX2 expression, which is transcriptionally regulated by NF κ B, acting alone or together with TonEBP/NFAT5 transcription factor 14. TonEBP/NFAT5 also regulates XBP1 expression 7. Thus, we hypothesized that XBP1s might be involved in COX2 expression. As seen in Figure 3, the sharp increase of *Ptgs2* mRNA (encoding COX2 protein) induced by hypertonicity was blocked by 4 μ 8C (Figure 3**A**), indicating that XBP1 maturation is required for COX2 transcription. Unpredictably, and despite a significant decrease in *Ptgs2* mRNA, after 4 μ 8C treatment we found an increase in COX2 protein level (Figure 3**B**, p < 0.001). Considering that *Ptgs2* mRNA levels were decreased, the accumulation of COX2 protein may suggest an impairment in protein degradation. To evaluate this possibility, we performed a cycloheximide (CHX)-chase assay. Cells treated with 4 μ 8C maintained significantly higher COX2 protein levels compared to control cells: 80% vs 51 % at 6 h (p < 0.01) and 64% vs 40% at 9 h (Figure 3**C**, p < 0.01). These results show that COX2 degradation is slowed down when the IRE1 α -XBP1s pathway of the UPR is impaired. To determine the subcellular localization of COX2 in cells treated 4 μ 8C, we evaluated COX2 distribution by confocal microscopy using calnexin as an ER marker (Figure 4**A**). Renal epithelial cells cultured in hypertonic conditions (upper panels) exhibited a typical compartmentalization since COX2-specific signal was distributed in the perinuclear region and in the ER spread throughout the cytoplasm with a dotted pattern, co-distributing with calnexin-specific signal. Consistent with the results obtained by western blot analysis (Figure 3**B**), cells treated with 4 μ 8C (lower panels) displayed a high level of COX2-specific fluorescence, distributed around the nucleus and in cytoplasmic granules, resembling the pattern observed for calnexin labeling. Fluorescence colocalization was analyzed in a selected region (Figure 4**A**, dotted box, and Figure 4**B**). Line profile analysis (Figure 4**B**, white line, and Figure 4**C**) revealed overlapping fluorescence intensities for COX2 and calnexin. The colocalization of COX2 and calnexin was confirmed by Pearson correlation coefficient (r = 0.719) and overlapping Manders coefficient (M = 0.807) calculated in the region of interest. Therefore, the lack of mature *Xbp1(s)* mRNA is responsible for COX2 accumulation within the ER.

We next evaluated whether the decreased XBP1s activity affects PLA ${}_{2}$ and COX activities. To do this, two cellular pathways were evaluated: (1) Lands’ cycle, assessed by measuring the incorporation of ${}^{3}$ H-arachidonic acid ( ${}^{3}$ H-AA) into the *sn*-2 position of the glycerol backbone, and (2) the levels of PGE2 synthesized and released to the incubation medium. As seen in Figure 4**D**, pre-treatment with 4 μ 8C significantly reduced ${}^{3}$ H-AA incorporation into GP (p < 0.005), but significantly increased the level of PGE2, the main COX product in our system 19 (Figure 4**E**, p < 0.005). These results suggest an increase in AA release from membrane GP, likely resulting from upregulation of PLA ${}_{2}$ activity. Altogether, these results indicate that renal cells respond to osmotic stress by activating the IRE1 α -XBP1s–NF- κ B signaling pathway, with NF- κ B mediating the expression of the osmoprotective genes *Pla2g4a* and *Ptgs2.* The absence of a functional UPR not only impairs the restoration of ER homeostasis but also appears to prevent the targeted degradation of COX2, which consequently accumulates within ER cisternae, thereby disrupting AA metabolic homeostasis.

### IRE1 α -XBP1s-NF κ B signaling constitutes a survival pathway necessary for the establishment of the osmotic-induced differentiated phenotype

**Figure 5:**
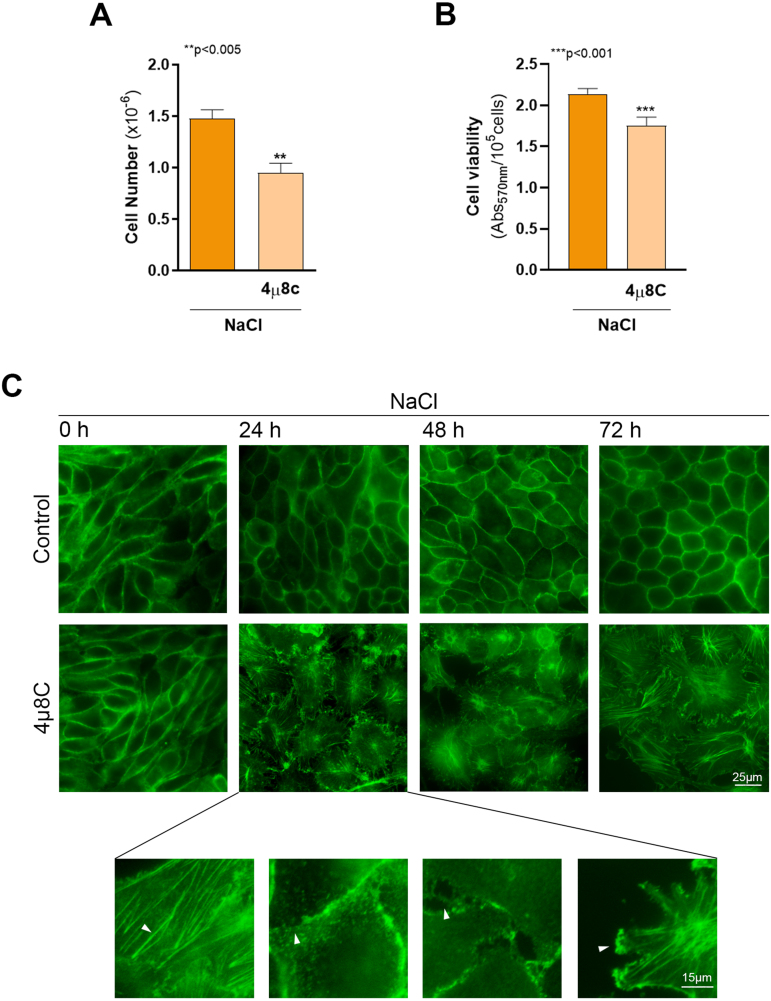
XBP1s downregulation prevents cell adaptation to osmotic stress. MDCK cells were cultured as described in Cell culture section and incubated for 24 h in hypertonic medium (NaCl) in the presence or in the absence of 20 μ M 4 μ 8C. **(A)** After treatments, cells were harvested and counted in a hemocytometer chamber. Results were expressed as cell number and represents the mean ± SEM of three independent experiments. **(B)** Cell viability was determined by Neutral Red uptake assay. Results were expressed as the absorbance measured at 570 nm normalized by cell number and represents the mean ± SEM of three independent experiments. Data were analyzed by unpaired two tailed Student’s t-test. ${}^{**}$ p < 0.005, ${}^{***}$ p < 0.001. **(C)** In another set of experiments, cells were seeded on glass coverslips and incubated for 0, 24, 48 and 72 h in hypertonic medium (NaCl) in the presence or in the absence of 20 μ M 4 μ 8C. F-actin was labeled by incubating samples with 3.3 μ g/mL phalloidin-FITC (green). Scale bar = 25 μ m. Lower panels show F-actin alterations observed in cells treated with 4 μ 8C for 24 h, indicated with white arrowheads: stress fibers, loss of cortical actin continuity, gaps, and lamellipodia-like structures, respectively. Fluorescence microscopy images (600X magnification) were processed with Image J. Scale bar = 15 μ m.

Consistent with the altered expression of the osmoprotective proteins cPLA2 and COX2, the inhibition of the IRE1 α -XBP1s-NF κ B signaling resulted in a reduction of 38% in cell number (Figure 5**A**) and 18% in cell viability (Figure 5**B**). The blockage of IRE1 α -XBP1s-NF κ B also affected hypertonic cell phenotype. After adaptation period (24 h), MDCK cells subjected to hypertonic culture conditions undergo a differentiation process between 24 h and 72 h, including cell polarization, the transition from a fibroblast-like phenotype to a cobblestone morphology, the establishment of cell-cell junctions, and the appearance of a primary cilium 20. Figure 5**C** shows that control cells subjected to hypertonic medium gradually progress from fibroblast-like morphology (0 h) to a well-established cobblestone phenotype that corresponds to differentiated cells (48–72 h). Pre-treatment with 4 μ 8C completely altered cell morphology at 24 h and prevented the acquisition of the typical cobblestone morphology of a differentiated monolayer. Moreover, XBP1s downregulation led to an increase in stress fiber formation, loss of cortical actin continuity and cell–cell contacts, and the emergence of lamellipodia-like structures (magnified lower panels).

**Figure 6:**
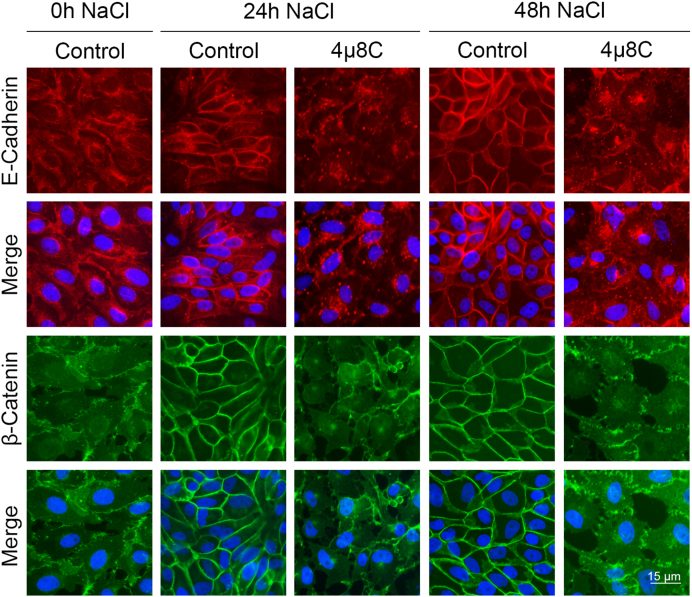
IRE1 α inhibition prevents hypertonic-induced MDCK polarization. MDCK cells were grown on glass coverslips and incubated in hypertonic medium (NaCl) for 0, 24 and 48 h in the presence or in the absence of 20 μ M 4 μ 8C. E-cadherin was visualized by using a mouse monoclonal anti-E-cadherin (1:50) primary antibody and donkey anti-mouse IgG Alexa Fluor 546-conjugated (1:200) antibody (red). β -catenin was visualized by using a mouse monoclonal anti- β -catenin (1:200) primary antibody and a goat anti-mouse IgG Alexa Fluor 488-conjugated (1:200) antibody (green). Nuclei were visualized with 2.5 μ M Hoechst 33258 (blue). Fluorescence microscopy images (600X magnification) were processed with Image J. Scale bar = 15 μ m.

Then, we analyzed cellular distribution of E-cadherin and β -catenin (two main components of adherens junctions, AJ) in the presence of 4 μ 8C (Figure 6). In isosmolar control conditions (0 h), E-cadherin was distributed throughout the cytoplasm and concentrated in perinuclear granules, whereas β -catenin exhibited a cytoplasmic pattern with a faint nuclear signal. Hypertonicity induced a gradual relocalization of the two proteins, increasing their localization at the cell periphery (NaCl 24 h control panels) to establish continuous belt-like structures characteristic of a polarized epithelium (NaCl 48 h control panels). The inhibition of XBP1s mRNA maturation prevented the distribution of E-cadherin and β -catenin to the cell periphery after 24 h and 48 h. Additionally, under these conditions, E-cadherin increased its localization in multiple cytoplasmic granules, whereas β -catenin became concentrated in perinuclear structures with a faint nuclear signal. These results suggest that the absence of XBP1s impedes the establishment of proper cell–cell interactions, consequently leading to a loss of cellular polarization.

### PGE2 contributes to the establishment of cell-cell junctions

**Figure 7:**
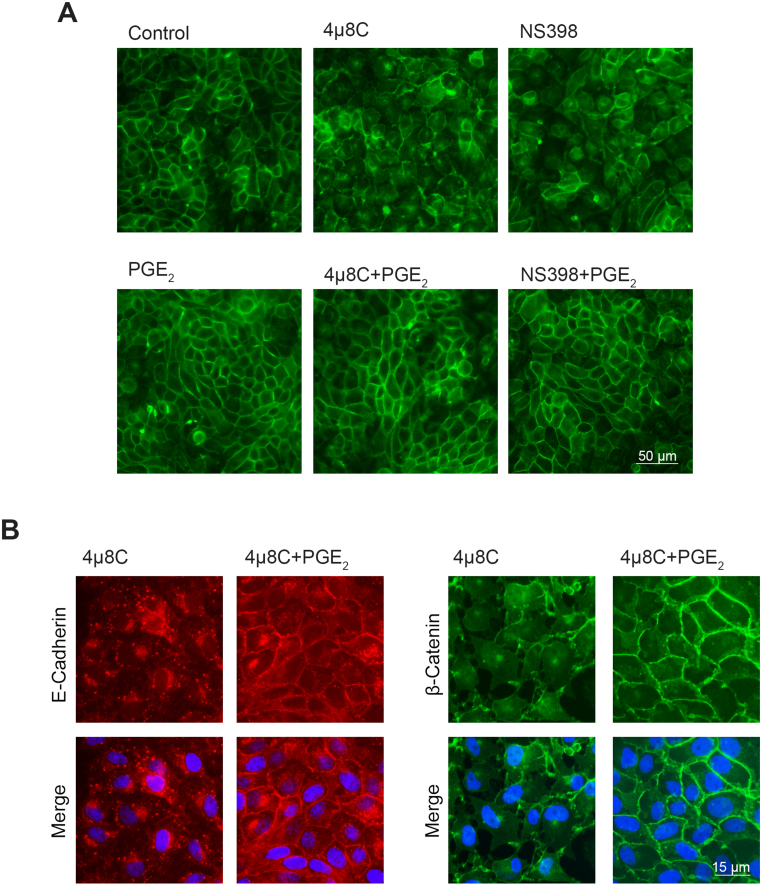
PGE2 contributes to the establishment of cell-cell junctions. MDCK cells were seeded on glass coverslips and cultured as described in Cell culture section **(A)** In one set of experiments, cells were incubated for 24 h in hypertonic medium alone (control) or with the adding of 20 μ M 4 μ 8C, 10 μ M NS398, 10 μ M PGE ${}_{2}$ , 20 μ M 4 μ 8C plus 10 μ M PGE ${}_{2}$ , or 10 μ M NS398 plus 10 μ M PGE ${}_{2}$ . F-actin was labeled by incubating samples with 3.3 μ g/mL phalloidin-FITC (green). Fluorescence microscopy images (200X magnification) were processed with Image J. Scale bar = 50 μ m. **(B)** In other set of experiments, cells were incubated for 24 h in hypertonic medium with 20 μ M 4 μ 8C or 20 μ M 4 μ 8C plus 10 μ M PGE2. E-cadherin was visualized by using a mouse monoclonal anti-E-cadherin (1:50) primary antibody and donkey anti-mouse IgG Alexa Fluor 546-conjugated (1:200) antibody (red). β -catenin was visualized using a mouse monoclonal anti- β -catenin (1:200) primary antibody and a goat anti-mouse IgG Alexa Fluor 488-conjugated (1:200) antibody (green). Nuclei were visualized with 2.5 μ M Hoechst 33258 (blue). Fluorescence microscopy images (600X magnification) were processed with Image J. Scale bar = 15 μ m.

Our data show that the loss of XBP1s expression impedes the establishment of cell-cell interaction, hindering the acquisition of differentiation phenotype, and that affects cPLA ${}_{2}$ -COX2 axis, increasing PGE2 levels. To evaluate whether PGE2 participates in cell-cell adhesion assembly, we studied the effect of high (by adding exogenous PGE2) and low concentrations of PGE2 (by inhibiting COX2) on cell morphology (Figure 7**A**). The addition of exogenous PGE2 to control cells did not significantly change monolayer morphology when compared with its respective hypertonic control. As it was shown before, the treatment with 4 μ 8C completely altered cell morphology, but the addition of exogenous PGE2 prevented such phenotype. When COX2 activity was blocked with NS398, we found that the monolayer showed a similar altered morphology than 4 μ 8C-treated cells. Similarly, when PGE2 was exogenously added, morphological alterations were not observed. These results suggest that PGE2 plays a fundamental role in the establishment of cell-cell adhesion.

Figure 7**B** shows the distribution of E-cadherin and β -catenin in cells cultured in the presence of 4 μ 8C alone or with exogenous PGE2. In the presence of 4 μ 8C, E-cadherin is accumulated in granules near the nuclear region. The addition of PGE2 allowed the localization of E-cadherin to the cell periphery. However, some granules were still observed near the nuclei, suggesting that PGE2 exerts its effects even though ER stress is not fully resolved. Similar observations were found when β -catenin was analyzed. When cells were cultured with 4 μ 8C alone, β -catenin was accumulated in a vesicle-like structure near the nuclei. The addition of PGE2 enhanced β -catenin localization at the cell surface (Figure 7**C**). These data support the hypothesis that PGE2 contributes to hypertonic-induced MDCK polarization by facilitating AJ assembly.

## DISCUSSION

The tubular structures that comprise renal inner medulla are physiologically immersed in a hyperosmolar ECM. The osmolarity in this kidney zone can constantly vary depending on the hydric state of the organism, and it is well described that the cell possesses various mechanisms to adapt and survive against osmolarity changes 2. We demonstrated that the maintenance of membrane homeostasis through GP synthesis and fatty acid remodeling contributes to the adaptation and survival under hypertonic stress 3. We also showed that PG (metabolites of the AA released from membrane GP) greatly contribute to the maintenance of membrane homeostasis since they regulate renal GP synthesis 11. In recent work, we showed that the IRE1 α -XBP1s 7 and the cPLA2-COX2 10 pathways participate in the upregulation of GL metabolism when cells are subjected to osmotic stress. Both pathways contribute to membrane homeostasis, osmoadaptation, and epithelial differentiation. In addition, we demonstrated that hypertonic induction of COX2 expression is driven by a synchronized action of TonEBP and NF κ B 14. On the other hand, some authors reported that ER stress UPR activation modulates NF κ B pathway 15. Hence, we propose NF κ B as the central nexus between the UPR and COX2, two osmoprotective pathways. We evaluated the crosstalk between UPR and AA metabolism in renal epithelial cells subjected to hypertonicity. Our data demonstrate that both pathways must necessarily be in contact in response to hypertonic stress to allow cell osmoprotection.

The reported mechanism for NF κ B activation through IRE1 α consists of the recruitment of TNF Receptor Associated Factor 2 (TRAF2) complex by IRE1 α kinase, which associates and activates I κ B kinase (IKK), promoting NF κ B nuclear translocation by a non-canonical pathway 21, 22. This is not the case of our system, since 4 μ 8C inhibits IRE1 α endoribonuclease but not its kinase-associated activity 23. Our data show that hypertonicity-mediated NF κ B activation depends on XBP1s expression (Figure 1), consistent with findings reported by Rao et al., who demonstrated that XBP1s facilitates p65 nuclear translocation in macrophages by negatively regulating metallothionein 2 expression 24.

AA is a key fatty acid that can be transformed into different lipid mediators by the action of enzymatic systems. In the kidney and renal cell lines, the conversion of AA into PG is a main metabolic pathway since PG mediate numerous renal homeostatic functions. The first step in the activation of AA metabolism is the release from membrane GP by the action of enzymes with PLA ${}_{2}$ activity. The mammalian PLA ${}_{2}$ superfamily comprises more than 50 enzymes classified into several groups and families based on structural and biochemical characteristics 25. Among them, cPLA ${}_{2}$ stands out due to its marked preference for GP containing AA at the *sn*-2 position as substrates 26. Zablocki et al. first demonstrated that hypertonicity increases PLA ${}_{2}$ activity in renal medulla and MDCK cells 27. We recently showed that the hypertonicity-induced increase in AA release is mediated by cPLA ${}_{2}$ activity and is, at least in part, a consequence of increased mRNA and protein expression 10. Although the 5’-flanking region of the *Pla2g4a* gene exhibits features typical of a housekeeping gene 28, and despite it is widely accepted that cPLA ${}_{2}$ activity is primarily regulated at post-transcriptional and post-translational levels 16, our results demonstrate that hypertonicity induces the transcriptional upregulation of cPLA ${}_{2}$ in renal epithelial cells (Figure 2**A**). We also showed that NF κ B is responsible for the hypertonicity-induced cPLA ${}_{2}$ upregulation (Figure 2**B** and **C**). This finding is consistent with the fact that a κ B response element is present in *Pla2g4a* promoter 17. The downregulation of XBP1s prevented hypertonic upregulation of cPLA ${}_{2}$ (Figure 2**D** and **E**), strongly suggesting the involvement of the IRE1 α -XBP1s cascade in the subsequent NF κ B-mediated transcription of cPLA ${}_{2}$ .

We previously showed that, under osmotic stress, NF κ B upregulates COX2 expression 9. NF κ B pathway can be activated by ER stress-UPR signaling. Rasheed et al. reported that ER stress induces the expression of COX2 and increases PGE2 synthesis via p38-MAPK and NF κ B signaling 29. In renal cells, the induction of ER stress-UPR signaling has been widely reported in diabetic nephropathy 30. However, there are almost no reports about renal hyperosmolarity and ER stress. We previously showed that hypertonicity induces ER stress 7. Herein we demonstrate for the first time that IRE1 α ribonuclease inhibition halts hypertonicity-induced *Ptgs2* transcription (Figure 3**A**) allowing us to propose that the IRE1 α -XBP1s-NF κ B signaling pathway regulates the transcription of the *Ptgs2* gene. However, we cannot discard that XBP1s may directly activate the transcription of cPLA ${}_{2}$ and/or COX2 proteins. In this context, Chopra et al. reported the presence of UPR-A and X-box elements in the regulatory region of the *Ptgs2* gene 13. On the contrary, we did not identify any XBP1s binding site within the regulatory region of *Pla2g4a* gene (not shown).

Despite the decrease in *Ptgs2* mRNA, the downregulation of XBP1s led to a marked increase in COX2 protein levels (Figure 3**B**). As it’s shown COX2 accumulation was a consequence of a decrease in protein degradation (Figure 3**C**). COX2 is synthesized by ER-associated ribosomes and localizes to the inner hemilayer of the ER and the outer membrane of the nuclear envelope 31. For entry into ER-associated degradation (ERAD), COX2 must be N-glycosylated in the ER lumen at the Asn-594 residue within its instability motif 32. Based on this, we hypothesized that the osmotic-induced loss of ER homeostasis impedes the proper modification of COX2 protein for degradation. Our results show that IRE1 α inhibition extends COX2 half-life, leading to its accumulation within the ER (Figure 3**C** and 4**A**). Despite this, COX2 was still active and synthesized PGE2.

Regarding the overall effect of IRE1 α inhibition on the cPLA ${}_{2}$ -AA-COX2-PG pathway, our results show that XBP1s downregulation reduces AA incorporation into GP (Figure 4**D**). For a new AA molecule to be incorporated into GP, a PLA ${}_{2}$ must first act by releasing the fatty acid esterified at the sn-2 position, thereby generating a lysophospholipid (LisoGP) molecule. Free AA is activated to arachidonyl CoA by means of ACSL4 enzyme and then esterified at the *sn-2* position of the LisoGP, contributing to GP turnover through Lands’ cycle. Thus, the decrease observed in AA incorporation to GP is probably due to an increase in the activity of the cPLA ${}_{2}$ and/or other PLA ${}_{2}$ (sPLA ${}_{2}$ or iPLA ${}_{2}$ ). Apart from COX, the released AA can be oxidized by LOX or CYP450 enzymes to generate different oxylipins 33. Considering the decreased expression of cPLA ${}_{2}$ mRNA and protein when IRE1 α -XBP1 is blocked, and the importance of PGE2 for the maintenance of epithelial structure, we could speculate that all AA released by the remaining PLA ${}_{2}$ activity is driven to PGE2 biosynthesis and not to the other pathways. In addition, it could be possible that LPLAT activity was decreased, thus impeding the incorporation of AA to GP to close Lands’ cycle. However, these complex hypotheses must be proven. The increased synthesis of PGE2 indicates that at least a fraction of ER-associated COX2 retains its catalytic activity.

In line with its aforementioned role in cell adaptation and survival to osmotic stress, AA metabolism disruption notably altered cell viability (Figure 5**A** and **C**). Moreover, it dramatically altered cell morphology, preventing the acquisition of the typical cobblestone-like differentiated phenotype (Figure 5**C**). In cell culture, following a 24 h adaptation period to hypertonicity, MDCK cells undergo a differentiation process that includes cell polarization, characterized by the assembly and maturation of cell-cell junctions and the establishment of well-defined apical and basolateral plasma membrane domains 34.

As we show herein, inhibition of the UPR alters AA metabolism, which is required to maintain lipid homeostasis. Therefore, hypertonicity-induced ER stress remains unresolved, leading to intracellular accumulation of E-cadherin and β -catenin, thereby impairing AJ assembly and cell polarization (Figure 6). In agreement with our findings, Geng et al. reported that thapsigargin-induced ER stress promotes O-linked β -N-acetylglucosamine modification of the cytosolic domain of E-cadherin at the ER and inhibits its proteolytic cleavage in the trans-Golgi network, thus preventing E-cadherin trafficking to the plasma membrane 35. Furthermore, Pallet et al. demonstrated that cyclosporin induces ER stress, which drives dedifferentiation of normal human renal epithelial cells through downregulation of E-cadherin and the translocation of β -catenin from the AJ to the cytoplasm and nucleus 36. Similarly, Dickhout et al. showed that ER stress disrupts epithelial junctions in HK-2 cells and induces β -catenin translocation to perinuclear structures where it colocalizes with the ER marker BiP 37.

Our work also demonstrates that the impaired cell-cell adhesion assembly was a consequence of unresolved ER stress, due to the decrease in the activity of the IRE1 α -XBP1s branch of the UPR. In this context, our results indicate that the increase in PGE2 production in 4 μ 8C-treated cells is associated with a protective action rather than cell morphology alterations (Figure 7). In physiological conditions, PGE2 plays important roles participating in the regulation of tubular water and sodium transport, glomerular filtration, and vascular resistance. All these renal functions are regulated by PGE2 via its EP receptors. Changes in renal medullary interstitial osmolarity increase PGE2 levels, upregulating COX2 expression, that exerts its protective effect by EP4 receptor increasing cAMP 38. Under osmotic stress, the activation of EP2/EP4 by PGE2 activates cAMP/PKA pathway leading to an increase in survivin and a decrease in Bad-induced apoptosis by impeding Bad phosphorylation 39, 40.

Apart from the canonical PGE2-EP2/EP4 pathway, other signaling pathways can be activated. It is widely reported that cAMP is a key regulator of cell-cell adhesion in endothelium, epithelial cells and cardiomyocytes. Various experimental models indicate that endothelial AJ cadherins, keratinocyte desmosomes, and cardiomyocyte intercalated discs are primary targets within a complex regulatory framework activated by cAMP 41. In this function, cAMP could act through its typical pathway or by activating an A kinase anchoring protein (AKAP). Thus, cAMP leads to the dissociation of the PKA catalytic - regulatory protein complex. AKAP proteins, usually bound to the cytosolic domain of cadherins, attach to the regulatory subunits of PKA and drive its compartmentalization linking PKA to the classical cadherins VE-cadherin and E-cadherin 42, 43. In addition, cAMP activates exchange protein activated by cAMP (EPAC), that in turn activates the downstream GTPase Rap1, which is involved in the regulation of integrin-based focal adhesion assembly and promotes the establishment of ZO1-containing junctions 44. Moreover, it has been reported that PGE2 induces the expression of intercellular adhesion molecule-1 after EP4 activation via cAMP/EPAC/NF κ B signaling 45. Miyoshi et al. described that PGE2 promotes intestinal repair through epithelial stem cell differentiation 46, whereas Zang et al. reported that PGE2 inhibits epithelial to mesenchymal transition induced by hepatocyte growth factor in MDCK cells 47. Other authors demonstrated the regenerative effect of high concentrations of PGE2 administered to a murine model of acute kidney injury AKI through Hippo/Yap 48 and Wnt4 pathways 49. Our experiments indicate that the PGE2 allows restoration of cell-cell junctions, acting as a cytoprotective molecule. However, we cannot assess the signaling pathway involved in this effect and will be studied in our laboratory.

In our system, we showed that the addition of PGE2 partially prevented morphological changes induced by XBP1s downregulation (Figure 7**A**), also suggesting that PGE2 has an important role in the establishment of the epithelial phenotype. These results are in line with our recent report demonstrating that PGE2 participates in epithelial restitution after calcium oxalate damage 12. Additionally, we showed that PGE2 facilitates E-cadherin and β -catenin trafficking to the cell surface (Figure 7**B**), demonstrating that PGE2 is implicated in hypertonicity-induced MDCK differentiation by promoting AJ assembly and cell polarization.

**Figure 8:**
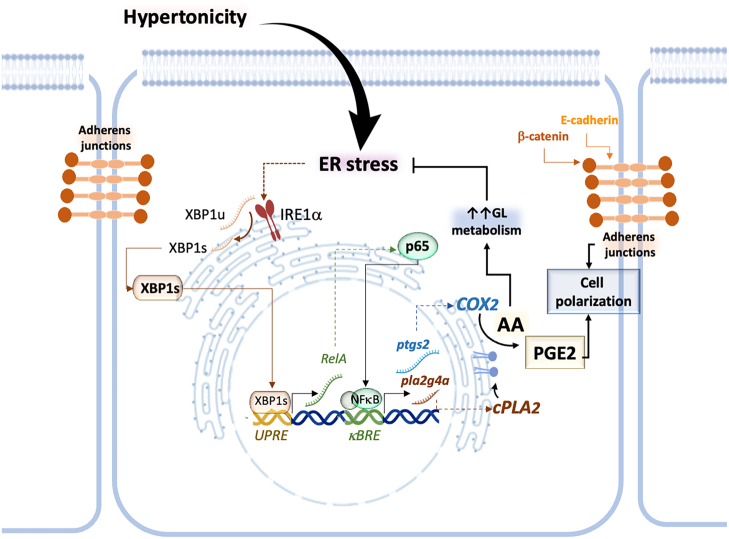
The role of IRE1 α -XBP1s-NF κ B signaling in osmoadaptation and renal epithelial cell differentiation. Hypertonicity induces ER stress, leading to the activation of IRE1 α RNase activity and the splicing of *XBP1(u)* into *XBP1(s)*, which is translated into the XBP1s protein. XBP1s transcriptionally activates the *RelA* gene, leading to p65-NF κ B protein expression, which mediates hypertonicity-induced cPLA2 and COX2 expression. The cPLA2–COX2 axis activates AA metabolism. cPLA2 releases AA from GP, which can promote GL synthesis and accumulation within lipid droplets, contributing to the membrane homeostasis and the alleviation of ER stress. Simultaneously, COX2 converts AA into PGE2, which facilitates E-cadherin and β -catenin trafficking to the cell surface, thereby promoting adherens junction (AJ) assembly and the restoration of renal epithelial cell polarization.

Altogether, the results presented in this work identify the IRE1 α -XBP1s-NF κ B axis as a central signaling in the regulation of hypertonic activation AA metabolism, providing a mechanistic framework to understand how renal cells coordinate stress adaptation with epithelial differentiation (Figure 8). Beyond the possible cellular mechanisms described here, our findings have significant physiological and pathophysiological implications. In the renal medulla, chronic stress is driven by extreme hypertonicity and hypoxia, which induces UPR-mediated lipid homeostasis to prevent cell death 7. The balance between the UPR pathways: the adaptive one, which promotes cell survival, and the apoptotic one, where the UPR response is excessive and cell death is activated, plays a critical role in determining the cell fate in ER stress 50, 51. In AKI, Li et al demonstrated that the activation of the IRE1 α -XBP1s axis is a critical regulator of autophagy and the pathway exerts a beneficial effect in damaged renal tubular epithelial cells during ischemic stress, suggesting that pharmacological modulation of this pathway may offer therapeutic avenues for preventing or mitigating AKI 52. Moreover, the inability to activate the UPR could maintain tubular cells in a dedifferentiated state, promoting the secretion of profibrotic factors and driving the AKI to chronic kidney disease transition through interstitial fibrosis 53. Thus, the IRE1 α -XBP1s pathway emerges as a potential translational target to ensure functional recovery and prevent long-term renal architectural decay.

## MATERIAL AND METHODS

### Materials and reagents

DMEM/Ham’s F12 (1:1) mixture, penicillin–streptomycin (10,000 U/mL), trypsin-EDTA solution, Hoechst 33258, ethidium bromide, goat polyclonal anti-rabbit IgG HRP conjugated, donkey polyclonal anti-mouse IgG Alexa Fluor® 488, donkey polyclonal anti-mouse IgG Alexa Fluor® 546 and donkey polyclonal anti-rabbit IgG Alexa Fluor® 546 conjugated secondary antibodies were obtained from Thermo Fisher Scientific (Waltham, MA, USA). Fetal bovine serum (FBS) was purchased from Natocor (Córdoba, Argentina). Bovine serum albumin (BSA), actinomycin D (AD), cycloheximide (CHX), phalloidin-FITC, 4 μ 8C, parthenolide (Parthe) and Neutral Red were obtained from Sigma-Aldrich (St. Louis, MO, USA). M-MLV retrotranscriptase, Oligo-dT primer, SV Total isolation System and Luciferase Assay System were obtained from Promega (Fitchburg, WI, USA). Master Mix qPCR sybr/ROX was purchased from Bio-Logicos products (Quilmes, Argentina). TransIT-X2® Dynamic Delivery System was purchased from MirusBio (Madison, WI, USA) Mouse monoclonal anti-cPLA ${}_{2}$ and mouse monoclonal anti β -Catenin antibodies were obtained from Santa Cruz Biotechnology (Santa Cruz, CA, USA). Goat polyclonal anti-mouse IgG HRP conjugated and goat anti-mouse IgG FITC conjugated antibodies were purchased from Jackson ImmunoResearch Laboratories (West Grove, PA, USA). Rabbit polyclonal anti-COX2 antibody, NS398 and prostaglandin E2 (PGE2) were obtained from Cayman Chemical (Ann Arbor, MI, USA). Mouse monoclonal anti-E-Cadherin, mouse monoclonal anti Calnexin and mouse monoclonal anti p65-NF κ B antibodies were purchased from BD Biosciences (Franklin Lakes, NY, USA). Donkey polyclonal anti-rabbit IgG HRP conjugated antibody, Polyvinylidene difluoride (PDVF) membranes, and ECL Plus Western blotting analysis system were obtained from GE Healthcare LifeSciences (Buckinghamshire, UK). Mouse monoclonal anti- β -Tubulin antibody was purchased from Biolegend (San Diego, CA, USA). TLC silica gel chromatoplates were obtained from Merck (Darmstadt, Germany). [5,6,8,9,11,12,14,15- ${}^{3}$ H(N)]-arachidonic acid ( ${}^{3}$ H-AA, spec. act. 62 Ci/mmol) were obtained from Perkin Elmer (Waltham, MA, USA). All general drugs and solvents used were obtained from Ciccarelli Laboratories (Santa Fe, Argentina) and were HPLC quality.

### Cell culture

Madin-Darby Canine Kidney (MDCK) cells were obtained from American Type Culture Collection (ATCC, CCL-34) and grown in a DMEM/Ham’s F12 (1:1) mixture containing 10% FBS, and 1% antibiotic mixture. After 24 h of culture (60-70% confluence), cells were placed in low-serum media (0.5% FBS) for 24 h and then subjected to hypertonic media (NaCl conditions) for different periods of time (0–72 h) by adding 125 mM NaCl to culture media to achieve a final osmolality of 520 ± 12 mOsm/kg H ${}_{2}$ O. In some experiments, commercial media without adding NaCl was used as isoosmolal control condition (298 ± 19 mOsm/kg H ${}_{2}$ O). Cultures were maintained at 37 ${}^{\circ }$ C in a humidified atmosphere with 5% CO ${}_{2}$ . All experiments were performed with mycoplasma-free cells (passages 57–59).

MDCK cells exhibit both morphological and functional characteristics of collecting ducts cells 54–56. Additionally, under hypertonic conditions, MDCK cells develop a differentiated phenotype, marked by the maturation of cell–cell junctions and the formation of a primary cilium 20. Consequently, this cell line serves as a valuable model for investigating the properties of the collecting duct.

### Treatments

To evaluate the role of XBP1s on arachidonic acid metabolism MDCK cells were treated with 20 μ M 4 μ 8C, a specific inhibitor of IRE1 α endoribonuclease activity involved in the unconventional splicing of *Xbp1u* mRNA 23. Under these experimental conditions, the conversion of *Xbp1u* to *Xbp1s* is completely blocked. To study the role of NF κ B in cPLA2 and COX2 expression, cells were treated with 3 μ M parthenolide, which inhibits I κ B kinase activity, preventing NF κ B activation 18. COX2 activity was inhibited with 2.5 μ M NS398. RNA transcription was inhibited by treating cells with 0.1 μ g/mL actinomycin D and protein translation was blocked with 25 μ M cycloheximide. Inhibitors were added 30 min before the addition of NaCl to the culture media to allow for uptake. To evaluate the effect of PGE2 on MDCK cell polarization and differentiation, we supplemented the culture medium with 10 μ M PGE2 30 min before the addition of NaCl. We chose this concentration based on a previous work from our laboratory, where we demonstrated that 10 μ M PGE2 was able to rescue the monolayer from the damage generated by calcium oxalate, restoring the polarized-differentiated phenotype of the renal epithelial cell monolayer 12.

After treatments, cells were washed with PBS and processed for fluorescence microscopy or treated with 0.25% trypsin-EDTA. Once cells were detached from plastic support, trypsin action was inhibited by adding 10% FBS. Total cell number and viability were assessed using a hemocytometer chamber in the presence of trypan blue 57. After counting, the cells were used in different experimental protocols as described below.

### RNA isolation and RT-qPCR assays

MDCK cells were cultured as described above. After treatments cells were harvested and total RNA was extracted utilizing the SV Total RNA Isolation System, according to the manufacturer’s instructions. First-strand cDNA was synthesized from the extracted RNA using MMLV reverse transcriptase and oligo-dT as a primer, mRNA levels of cPLA ${}_{2}$ and COX2 were measured by Real Time PCR (qPCR) on a StepOnePlus™ system (Thermo Fisher, Waltham, MA, USA) with Master Mix qPCR sybr/ROX. Specific primers were designed with PRIMER3 software (BioTools – University of Massachusetts Medical School). β *-actin* was used as housekeeping gene. Quantification was performed using the relative standard curve method. The sequences of primers used were: *Pla2g4a* 5’-GGTCGGATTCTCAGGTGTGA-3’ and 5’-ATGTGGAGCCA GAAAGACCA-3’; *Ptgs2* 5’-TCCCATCTGTTCACCTGACT-3’ and 5’-AGACTGGATTGAGGCAGTGT-3’*;* β *-actin* 5’-CAAAGCCAACCGTGAGAAG-3’ and 5’-C AGAGTCCATGACAAT ACCAG-3’. *RelA.*

To confirm the effect of 4 μ 8C on IRE1 α RT-PCR were performed using primers designed to amplify both *Xbp1(u)* and *Xbp1(s)*: 5’-ACCCTGGCTACTGAAGAGGA-3’ and 5’-TCAACGCTGTCAGAATCCTAT-3’. PCR products were resolved in 7% polyacrylamide gels containing ethidium bromide and evidenced under UV light.

### Western blot analysis

After treatments cells were collected as described. After counting, pellets were resuspended in lysis buffer (0.089% NaCl–phosphate buffer, pH 7.2, containing 0.05% Triton X-100) containing 1% ProteinSafe protease inhibitor cocktail (TransGen Biotech Co, Beijing, China). Total protein content was determined by Lowry method 58, aliquots from samples containing 20 μ g of proteins were mixed with 5X Laemmli buffer and incubated at 100 ${}^{\circ }$ C for 5 min. Proteins were separated on 10% SDS/PAGE gels and transferred to PVDF membranes. The membranes were then blocked for 90 min at room temperature in TBS buffer containing 1% Tween and 5% non-fat milk and incubated overnight at 4 ${}^{\circ }$ C with mouse monoclonal anti-cPLA ${}_{2}$ (1:250), rabbit polyclonal anti-COX2 (1:250), mouse monoclonal anti-p65-NF κ B (1:250) or mouse monoclonal anti- β -Tubulin (1:1000) antibodies. Blots were washed and incubated 90 min at room temperature with donkey polyclonal anti-rabbit IgG HRP conjugated antibody (1:5000) or goat polyclonal anti-mouse IgG HRP conjugated antibody (1:7500) secondary antibodies, depending on primary antibody utilized.

Bands were evidenced by means of ECL Plus Western blotting analysis system and chemiluminescence were detected on a Sapphire Biomolecular bioimager (Azure Biosystems, CA, USA). Densitometries were performed using GEL ANALYZER 2010 free software (developed by Istvan Lazar Jr., PhD and Istvan Lazar Sr., PhD, CSc).

### Reporter gene assay

MDCK cells were seeded in 48-well plates and grown as described above. After reaching 70% confluence, 0.2 μ g of NF κ B reporter plasmid (p κ B-Luc) was transfected using TransIT-X2® Dynamic Delivery System following manufacturer’s instructions. This plasmid was gently gifted by Dr Omar Coso (IFIBYNE-CONICET). Then, cells were incubated for 24 h in hypertonic conditions in the presence or in the absence of 20 μ M 4 μ 8C. Luciferase activity was measured using the Luciferase Assay System following manufacturer’s instructions. Results were expressed as relative luciferase activity units (RLU) and normalized by total protein content.

### Microscopy experiments

Cells were seeded on glass coverslips and cultured and treated as described in Cell culture and Treatments sections . After treatments, cells were fixed with 4% (w/v) paraformaldehyde in PBS for 15 min and permeabilized with 0.1% (w/v) Triton X-100 in PBS for 15 min. To evaluate cell morphology, F-actin was labeled by incubating samples with 3.3 μ g/mL phalloidin-FITC for 1 h at room temperature. After labeling, cells were washed with PBS and mounted with a drop of Vectashield mounting medium. For immunofluorescence experiments, after cell permeabilization, samples were washed with PBS and blocked with 3% BSA in PBS for 1 h at room temperature and then incubated overnight at 4 ${}^{\circ }$ C with primary antibodies: mouse monoclonal anti-p65-NF κ B (1:50), rabbit polyclonal anti-COX2 (1:75), mouse monoclonal anti-Calnexin (1:50), mouse monoclonal anti-E-Cadherin (1:50) or mouse monoclonal anti- β -Catenin (1:200). After that, samples were washed with PBS and incubated 1 h at room temperature with secondary antibodies: goat anti-mouse IgG Alexa Fluor 488-conjugated antibody (1:200), goat anti-rabbit IgG FITC-conjugated antibody (1:200), donkey anti-mouse IgG Alexa Fluor 546-conjugated antibody (1:200) or donkey anti-rabbit Alexa Fluor 546-conjugated antibody (1:200), and with 2.5 Hoechst 33258 to visualize nuclei. Then, samples were washed with PBS and mounted with a drop of Vectashield mounting medium.

Wide-field fluorescence images were obtained using a Nikon Eclipse Ti microscope (with Plan apo VC 60X and 20X objectives, Nikon, Tokyo, Japan) with acquisition software Micrometrics SE Premium (Accu-Scope, Commarck, NY, USA). Confocal microscopy images were obtained using a confocal Zeiss LSM900/Axio Imager.Z2 microscope (with a Plan APOCHROMAT 639 objective, Jena, Germany) with acquisition software ZEN BLUE v3.3 (Zeiss, Jena, Germany). Images were processed using IMAGE J v.1.54d software (Bethesda, MD, USA).

### Neutral red uptake assay

This assay is based on the capacity of lysosomes of uptaking and retaining Neutral Red colorant after its protonation. In viable cells, Neutral Red is trapped in lysosomes, while in nonviable cells with impaired proton transport, it is not retained 59. Cells were cultured in 48- or 96-well multiwell plates as described in Cell culture section , and after treatment, the media were removed, cells were washed with PBS and incubated with 40 μ g/mL Neutral Red solution in non-supplemented media for 120 min at 37C. After incubation, the retained Neutral Red was solubilized with ethanol:water:acetic acid (50:49:1, v/v) solution, and the absorbance in each sample was determined at 570 nm.

### Glycerophospholipid fatty acid turnover

This assay was performed to evaluate the role of XBP1s on GP fatty acid turnover and PLA ${}_{2}$ activity (Lands’ Cycle). To do this, GP were labeled with radiolabeled AA ( ${}^{3}$ H-AA). MDCK cells were seeded on 12-well plates and cultured under hyperosmolar conditions for 24 h as described above. Before harvesting at 24 h, 0.4 μ Ci/mL ${}^{3}$ H-AA was added to each well and incubated to steady-state (3 h). In this condition, cells uptake ${}^{3}$ H-AA, which is rapidly activated to ${}^{3}$ H-AA-CoA by the enzyme long-chain-fatty-acid-CoA ligase 4 (ACSL4) and subsequently can be acylated to GP and TG acylation (even though in a significantly lesser extent). Acylation within GP is a dynamic process involving the sequential action of PLA ${}_{2}$ , which releases the previously acylated fatty acid from the *sn*-2 position of the glycerol backbone, and acyl-CoA:lysophospholipid acyltransferase, which re-esterifies AA into *sn*-2 position of GP, in this case the ${}^{3}$ H-AA. Considering this cycle, we hypothesized that a decay in the incorporation of ${}^{3}$ H-AA into GP could be indicative of a reduction in PLA ${}_{2}$ activity, thereby impeding the liberation of the *sn-*2 position necessary for the esterification of AA. After incubation, cells were harvested, counted, and total lipids were extracted according to Bligh and Dyer method 60. Briefly, after counting cells were centrifuged, and pellets were resuspended in 0.4 mL of water, 1 mL of methanol and 0.5 mL of chloroform. The samples were vortexed gently for 30 s and incubated for 15 min on ice. Then, 0.5 mL of chloroform and 0.5 mL of water were added, samples were vortexed 30 s and centrifuged for 5 min at 800 g. The lower organic phase containing lipids was collected and dried under a nitrogen stream. Lipids were separated by TLC developed in a mixture of petroleum ether/n-hexane/ethylic ether/acetic acid (40:40:20:1, v/v) as a solvent system. The plates were exposed to iodine vapors to visualize lipid mass. Different lipid classes were identified by retention factors (Rf) 4. Spots corresponding to GP were scrapped and radioactivity incorporated was measured by liquid scintillation counting.

### PGE2 extraction, separation and quantification

Culture media containing PG were acidified to pH 3 by adding 1 M citric acid. The media were then mixed with two volumes of chloroform. After a brief centrifugation to separate the phases, the lower organic phase containing PG was isolated and dried under a stream of nitrogen. To determine PGE ${}_{2}$ levels, dried extracts were resuspended in 50 μ L of methanol formic acid 0.1% v/v and PGE ${}_{2}$ was quantified using a HPLC-MS/MS system consisting of an UltiMate 3000 HPLC coupled to a TSQ Quantum Access MAX Triple Quadrupole Mass Spectrometer with electrospray ionization (Massachusetts, USA). HPLC was performed using a BDS Hypersil C18, 100 × 2,1 mm, 2.4 μ m. The mobile phase consisted of MeOH:H ${}_{2}$ O Formic Acid 0.1% v/v using a gradient mode (0–5 min: 30% MeOH:70% H ${}_{2}$ O 0.1% formic acid; 12–15 min: MeOH 100%; 18-22: 30% MeOH:70% H ${}_{2}$ O 0.1% formic acid; Flow 0.3 mL.min-1; the run time was 22 minutes and the column temperature, and the sampler were set at 40 ${}^{\circ }$ C and 10 ${}^{\circ }$ C respectively. Injection volume was 5 μ L. MS/MS conditions were: spray voltage 4000, vaporizer temperature 270, capillary temperature 290, while sheath gas pressure and aux gas pressure were set at 10 and 35 units, respectively. Detection was performed in SIM and SRM mode. Transitions were 351,3 → 271,3 (25 eV) and 351,3 → 189,2 (30 eV).

### Statistical analysis

Statistical analysis was performed by using GraphPad Prism8 Software. Data were expressed as mean ± SEM. When two experimental groups were compared, data from control and treatments were analyzed using unpaired two tailed Student’s t-test. When more than two groups were compared, data from control and treatments were analyzed using one-way ANOVA followed by a posteriori Dunnett’s test. P values lower than 0.05 were considered statistically significant.

## CONFLICT OF INTEREST

The authors declare no conflict of interest.

## ABBREVIATIONS

AA – arachidonic acid

AD – actinomycin D

AJ – adherens junctions

CHX – cycloheximide

COX2 – cyclooxygenase 2

cPLA_2_ – cytosolic phospholipase A_2_

ECM – extracellular matrix

ER – endoplasmic reticulum

FA – fatty acids

GP – glycerophospholipids

LD – lipid droplets

MDCK – Madin-Darby Canine Kidney

PG – prostaglandins

PGE2 – prostaglandin E2

TG – triacylglycerides

UPR – unfolded protein response
